# Supplementary material for: Resolving the full spectrum of human genome variation using Linked-Reads
Source: Genome Res. 2019 Apr;29(4):635–45. doi: 10.1101/gr.234443.118 (PMC6442396; doi:10.1101/gr.234443.118)
Supplement: Supplemental Material [file supp_gr.234443.118_Supplemental_longranger-2.2.zip › longranger-2.2.tar.bz2!/lr2.2-public/lib/stan/cmdstan/stan/doc/api/html/hypergeometric_8hpp_source.html]

Stan: src/stan/prob/distributions/univariate/discrete/hypergeometric.hpp Source File


|  |  |
| --- | --- |
|  | Stan  2.5.0  probability, sampling & optimization |


- Main Page
- Related Pages
- Namespaces
- Classes
- Files

- File List
- File Members

All Classes Namespaces Files Functions Variables Typedefs Enumerations Enumerator Friends Macros Pages

- src
- stan
- prob
- distributions
- univariate
- discrete

hypergeometric.hpp

Go to the documentation of this file.

1 #ifndef STAN\_\_PROB\_\_DISTRIBUTIONS\_\_UNIVARIATE\_\_DISCRETE\_\_HYPERGEOMETRIC\_HPP

2 #define STAN\_\_PROB\_\_DISTRIBUTIONS\_\_UNIVARIATE\_\_DISCRETE\_\_HYPERGEOMETRIC\_HPP

3

4 #include <boost/math/distributions.hpp>

5 #include <stan/prob/distributions/univariate/continuous/uniform.hpp>

6 #include <stan/agrad/partials\_vari.hpp>

7 #include <stan/math.hpp>

8 #include <stan/math/error\_handling.hpp>

9 #include <stan/meta/traits.hpp>

10 #include <stan/prob/traits.hpp>

11 #include <stan/prob/constants.hpp>

12 #include <vector>

13

14 namespace stan {

15

16  namespace prob {

17

18  // Hypergeometric(n|N,a,b) [0 <= n <= a; 0 <= N-n <= b; 0 <= N <= a+b]

19  // n: #white balls drawn; N: #balls drawn;

20  // a: #white balls; b: #black balls

21  template <bool propto,

22  typename T\_n, typename T\_N,

23  typename T\_a, typename T\_b>

24  double

25  hypergeometric\_log(const T\_n& n, const T\_N& N,

26  const T\_a& a, const T\_b& b) {

27  static const char\* function = "stan::prob::hypergeometric\_log(%1%)";

28

29  using stan::math::check\_finite;

30  using stan::math::check\_bounded;

31  using stan::math::check\_greater;

32  using stan::math::check\_consistent\_sizes;

33  using stan::prob::include\_summand;

34

35  // check if any vectors are zero length

36  if (!(stan::length(n)

37  && stan::length(N)

38  && stan::length(a)

39  && stan::length(b)))

40  return 0.0;

41

42

43  VectorView<const T\_n> n\_vec(n);

44  VectorView<const T\_N> N\_vec(N);

45  VectorView<const T\_a> a\_vec(a);

46  VectorView<const T\_b> b\_vec(b);

47  size\_t size = max\_size(n, N, a, b);

48

49  double logp(0.0);

50  check\_bounded(function, n, 0, a, "Successes variable", &logp);

51  check\_greater(function, N, n, "Draws parameter", &logp);

52  for (size\_t i = 0; i < size; i++) {

53  check\_bounded(function, N\_vec[i]-n\_vec[i], 0, b\_vec[i],

54  "Draws parameter minus successes variable", &logp);

55  check\_bounded(function, N\_vec[i], 0, a\_vec[i]+b\_vec[i],

56  "Draws parameter", &logp);

57  }

58  check\_consistent\_sizes(function,

59  n,N,a,b,

60  "Successes variable","Draws parameter",

61  "Successes in population parameter",

62  "Failures in population parameter",

63  &logp);

64

65  // check if no variables are involved and prop-to

66  if (!include\_summand<propto>::value)

67  return 0.0;

68

69

70  for (size\_t i = 0; i < size; i++)

71  logp += math::binomial\_coefficient\_log(a\_vec[i],n\_vec[i])

72  + math::binomial\_coefficient\_log(b\_vec[i],N\_vec[i]-n\_vec[i])

73  - math::binomial\_coefficient\_log(a\_vec[i]+b\_vec[i],N\_vec[i]);

74  return logp;

75  }

76

77  template <typename T\_n,

78  typename T\_N,

79  typename T\_a,

80  typename T\_b>

81  inline

82  double

83  hypergeometric\_log(const T\_n& n,

84  const T\_N& N,

85  const T\_a& a,

86  const T\_b& b) {

87  return hypergeometric\_log<false>(n,N,a,b);

88  }

89

90  template <class RNG>

91  inline int

92  hypergeometric\_rng(const int N,

93  const int a,

94  const int b,

95  RNG& rng) {

96  using boost::variate\_generator;

97

98  static const char\* function = "stan::prob::hypergeometric\_rng(%1%)";

99

100  using stan::math::check\_bounded;

101  using stan::math::check\_positive;

102

103  check\_bounded(function, N, 0, a+b, "Draws parameter", (int\*)0);

104  check\_positive(function,N,"Draws parameter", (int\*)0);

105  check\_positive(function,a,"Successes in population parameter", (int\*)0);

106  check\_positive(function,b,"Failures in population parameter", (int\*)0);

107

108  boost::math::hypergeometric\_distribution<>dist (b, N, a + b);

109  std::vector<double> index(a);

110  for(int i = 0; i < a; i++)

111  index[i] = cdf(dist, i + 1);

112

113  double c = uniform\_rng(0.0, 1.0, rng);

114  int min = 0;

115  int max = a - 1;

116  int mid = 0;

117  while(min < max) {

118  mid = (min + max) / 2;

119  if(index[mid] > c)

120  max = mid;

121  else

122  min = mid + 1;

123  }

124  return min + 1;

125  }

126  }

127 }

128 #endif

stan::math::check\_bounded

bool check\_bounded(const char \*function, const T\_y &y, const T\_low &low, const T\_high &high, const char \*name, T\_result \*result)

**Definition:** check\_bounded.hpp:79

stan::math::check\_greater

bool check\_greater(const char \*function, const T\_y &y, const T\_low &low, const char \*name, T\_result \*result)

**Definition:** check\_greater.hpp:58

traits.hpp

uniform.hpp

error\_handling.hpp

stan::math::max

double max(const double a, const double b)

**Definition:** max.hpp:7

stan::length

size\_t length(const T &)

**Definition:** traits.hpp:159

stan::math::binomial\_coefficient\_log

boost::math::tools::promote\_args< T\_N, T\_n >::type binomial\_coefficient\_log(const T\_N N, const T\_n n)

Return the log of the binomial coefficient for the specified arguments.

**Definition:** binomial\_coefficient\_log.hpp:63

stan::math::check\_finite

bool check\_finite(const char \*function, const T\_y &y, const char \*name, T\_result \*result)

Checks if the variable y is finite.

**Definition:** check\_finite.hpp:52

partials\_vari.hpp

constants.hpp

stan::prob::hypergeometric\_log

double hypergeometric\_log(const T\_n &n, const T\_N &N, const T\_a &a, const T\_b &b)

**Definition:** hypergeometric.hpp:25

stan::prob::hypergeometric\_rng

int hypergeometric\_rng(const int N, const int a, const int b, RNG &rng)

**Definition:** hypergeometric.hpp:92

traits.hpp

stan::prob::include\_summand

Template metaprogram to calculate whether a summand needs to be included in a proportional (log) prob...

**Definition:** traits.hpp:35

math.hpp

stan::prob::uniform\_rng

double uniform\_rng(const double alpha, const double beta, RNG &rng)

**Definition:** uniform.hpp:339

stan::math::check\_consistent\_sizes

bool check\_consistent\_sizes(const char \*function, const T1 &x1, const T2 &x2, const char \*name1, const char \*name2, T\_result \*result)

**Definition:** check\_consistent\_sizes.hpp:13

stan::max\_size

size\_t max\_size(const T1 &x1, const T2 &x2)

**Definition:** traits.hpp:191

stan::math::check\_positive

bool check\_positive(const char \*function, const T\_y &y, const char \*name, T\_result \*result)

**Definition:** check\_positive.hpp:54

stan::math::size

int size(const std::vector< T > &x)

**Definition:** size.hpp:11

stan::math::min

double min(const double a, const double b)

**Definition:** min.hpp:7

stan::VectorView

VectorView is a template metaprogram that takes its argument and allows it to be used like a vector...

**Definition:** traits.hpp:275

stan::math::dist

double dist(const std::vector< double > &x, const std::vector< double > &y)

**Definition:** dist.hpp:11


---

[ Stan Home Page ]

*© 2011–2014,
Stan Development Team.*
